# The Pathophysiology of Eosinophilic Esophagitis

**DOI:** 10.3389/fped.2014.00041

**Published:** 2014-05-30

**Authors:** Mayumi Raheem, Steven T. Leach, Andrew S. Day, Daniel A. Lemberg

**Affiliations:** ^1^School of Women’s and Children’s Health, University of New South Wales, Sydney, NSW, Australia; ^2^Department of Pediatrics, University of Otago (Christchurch), Christchurch, New Zealand; ^3^Department of Gastroenterology, Sydney Children’s Hospital, Sydney, NSW, Australia

**Keywords:** eosinophilic esophagitis, celiac disease, pathophysiology

## Abstract

Eosinophilic esophagitis (EoE) is an emerging disease characterized by esophageal eosinophilia (>15eos/hpf), lack of responsiveness to acid-suppressive medication and is managed by allergen elimination and anti-allergy therapy. Although the pathophysiology of EoE is currently unsubstantiated, evidence implicates food and aeroallergen hypersensitivity in genetically predisposed individuals as contributory factors. Genome-wide expression analyses have isolated a remarkably conserved gene-expression profile irrespective of age and gender, suggesting a genetic contribution. EoE has characteristics of mainly T_H_2 type immune responses but also some T_H_1 cytokines, which appear to strongly contribute to tissue fibrosis, with esophageal epithelial cells providing a hospitable environment for this inflammatory process. Eosinophil-degranulation products appear to play a central role in tissue remodeling in EoE. This remodeling and dysregulation predisposes to fibrosis. Mast-cell-derived molecules such as histamine may have an effect on enteric nerves and may also act in concert with transforming growth factor-β to interfere with esophageal musculature. Additionally, the esophageal epithelium may facilitate the inflammatory process under pathogenic contexts such as in EoE. This article aims to discuss the contributory factors in the pathophysiology of EoE.

## Introduction

Eosinophilic esophagitis (EoE) is an emerging condition characterized by severe isolated eosinophilic infiltration of the esophageal mucosa (1). It is unresponsive to acid-suppressive medication but responsive to the removal of dietary antigens and steroid anti-inflammatory medications (2). The identification of EoE as a disease occurred following investigations into treatment resistant patients with gastro-esophageal reflux disease (GERD) (3). The similarity in presentation of EoE and GERD necessitates the correlation of clinical and pathological findings and the establishment of diagnostic criteria differentiating the two diseases, to ensure accurate diagnosis and management (4).

The most recently updated consensus recommendations from The First International Gastrointestinal Research Symposium (FINGERS) state that EoE is a chronic clinicohistopathological disorder characterized by a myriad of symptoms distinct and similar to those of GERD (5). Clinically, EoE symptomatology varies with age, but is characterized by symptoms suggestive of esophageal dysfunction including dysphagia, abdominal pain, and episodes of food impactions. Children typically present with failure to thrive, vomiting, and heartburn (6).

Research has also identified specific endoscopic and histologic features more characteristic of EoE than GERD. Endoscopic findings such as rings, strictures, narrowed esophagus, linear furrows, crepe-paper mucosa, and white plaques along with histological findings such as maximal eosinophil counts greater than 15 eosinophils per high-powered-field (hpf), eosinophil microabscesses, eosinophil degranulation, spongiosis, and subepithelial fibrosis are more common in EoE patients than those with GERD (6, 7).

However, emerging evidence questions the reliability of using eosinophil counts as diagnostic of EoE. Some cases of GERD also meet these criteria of eosinophil numbers, indicating that this feature alone would introduce error in distinguishing EoE from GERD (6). While historically, unresponsiveness to acid-suppression therapy with high esophageal eosinophil density was pathognomonic of EoE, studies now show molecular and histological features that differentiate EoE from GERD (6). Increased awareness of features that define EoE has allowed more accurate diagnoses and enhanced management of patients.

Frequently, but not always, patients presenting with EoE have a history of food (8) or aeroallergen (9) hypersensitivity, elevated serum immunoglobulin (Ig)E, and responsiveness to diet restriction (8, 10) or anti-allergy therapy (11, 12). However, some patients with EoE have normal IgE levels. While the documented cytokine expression profile in the esophageal tissue in patients with EoE is that of a T_H_2 inflammatory response (13), T_H_1 cytokines have also been shown to be up-regulated in some patients. The later favors a type IV hypersensitivity (cell mediated) mechanism, and may explain the non-IgE cases of EoE (14).

This article aims to discuss the current understanding of the roles of eosinophils, mast cells, cytokines, chemokines, and esophageal epithelial cells, in the pathophysiology of EoE.

## Epidemiology

A number of studies have determined the prevalence of EoE in selected populations: some have suggested an increasing incidence of disease. The estimated prevalence of EoE in a pediatric population in Hamilton County, Ohio between 2000 and 2003, was ~4 in 10,000 with an incidence of 0.9–1.3 in 10,000 new cases per year (15). A 16-year long study on an adult Swiss cohort observed similar prevalence (~2/10,000) and incidence (1.4/100,000) rates (16). However, despite the increase in esophagogastroduodenal biopsies between 1982 and 1999, a retrospective study examining 666 patients found the incidence of EoE to be relatively stable during this time period (16). As such enhanced disease recognition rather than true increase in disease incidence may be contributing to the emergence of EoE as a high prevalence clinicopathologic entity.

Case reports of EoE are mainly from industrialized countries such as Australia, North America, Europe, and East Asia, with isolated reports from South America. Reports from African-American populations have been scarce (16, 17). EoE predominates in Caucasian (81%) (18) middle aged (30–40 years) men (72%) (17), although cases have been reported from all ages and ethnic backgrounds (19, 20). This observed higher disease rate in Caucasians compared to other ethnicities raises questions about race as a confounding factor in the pathophysiology of EoE. In a recent study comparing the clinical presentations of adult patients with EoE, Sperry et al. (17), suggested that African-American patients were younger at diagnosis, were more likely to present with failure to thrive and less likely to have esophageal rings than Caucasian patients. Whether the lower prevalence of the African-American population with EoE is attributable to misdiagnosis due to currently unknown presentations or a true lower prevalence, is uncertain. More research is required to fully establish the role of ethnicity in the pathophysiology of EoE.

Research showing seasonal variations of EoE have postulated that environmental factors may modify the presentation of EoE. Some investigators have reported an increase in the incidence of EoE during spring and summer seasons (21). However, these findings are controversial. Hurrel et al. (22) found a high prevalence of esophageal eosinophilia in adult patients living within the colder geographical climates of the US compared to those living in warmer areas. Other similar studies have also not supported seasonal variations (23).

## Genetic Heritability

Accumulating evidence has shown a strong familial association in EoE (15). Zink et al. (24) reported EoE to span over two generations in five out of seven families studied. In the same study, a longer term follow-up found a family history of EoE in 5 out of 30 patients with the condition. Concordantly, Noel et al. (15) found 6.8% of 103 pediatric patients with EoE to have a family history of dysphagia. EoE also exhibits a high sibling risk ratio (λ_S_), defined as the ratio of disease manifestation given that one’s sibling is affected, of ~80, compared to other atopic diseases such as asthma (λ_S_ ~ 2) (25). To allow comparison, inflammatory bowel disease, a condition known to run in families has a λ_S_ of 0.83 (26). Interestingly, recent studies have demonstrated the coexistence of EoE with another genetically inherited condition, celiac disease (CD) (27, 28), further highlighting a possible genetic contribution to EoE.

Several candidate genes for EoE have been identified. *CCL26/eotaxin-3 gene*, based on genome-wide association studies (GWAS), is the most highly expressed gene in EoE, being up-regulated between 50- and 100-fold in EoE patients (29). However, this disease-associated allele has only been found in 14% of cases (30), highlighting the contributions of other risk variants. More recently, Rothenberg et al. (29) identified and replicated a significant locus at 5q22.1 in European cases of EoE. The two genes that map to this locus are *TSLP* and *WDR36* (29). TSLP encodes a cytokine similar to interleukin (IL)-7 produced in the thymus and peripheral tissues, and acts to regulate T_H_2 responses (31). WD36 is co-regulated with T-cell growth factor IL-2 and has been linked to glaucoma (32). It was postulated that the male predominance of EoE may be related to the TSLP receptor residing within the pseudoautosomal region 1 on the X and Y chromosomes (Xp22.3, Yp11.3) (29). A more recent study showed that polymorphisms in *TSLP* are risk factors for the development of EoE, independent of allergy status and phenotypes (33). The same study found an association between polymorphisms in the thymic stromal lipoprotein receptor (*TSLPR*) gene on Xp22.3/Yp11.3 and EoE in male participants, suggesting a mechanism for the male predominance of EoE. Moreover, primary esophageal epithelial cells were shown to express *TSLP* mRNA in response to toll-like receptor 3 signaling, suggesting a possible contribution of *TSLP* in the inflammation and proliferation occurring in EoE affected esophagi. Furthermore, in a genome-wide microarray expression analysis, Lu et al. (34) identified 32 miRNA specific for EoE. Of these, miRNA-21 and miRNA-223 were the most up-regulated in untreated EoE and were down-regulated upon corticosteroid administration.

Despite this strong evidence supporting the genetic basis of EoE pathogenesis, studies have shown that the familial pattern of inheritance of EoE shares an underlying pathogenesis with sporadic cases of EoE (35).

## The Pathophysiology of EoE

Genetic predisposition may deem an individual vulnerable to the environmental triggers resulting in EoE. Frequently, patients presenting with EoE have a history of food (8) or aeroallergen (9) hypersensitivity, elevated serum IgE, and responsiveness to diet restriction (8, 10) or anti-allergy therapy (11, 36). Food hypersensitivity has been reported in 19–73% of children and 13–25% of adults with EoE (37). The reason for lower rates of food hypersensitivity in adults is unclear, but this feature may mean that adults are less responsive to diet restriction (8). Regardless, EoE is considered an immunoallergenic disorder, whereby esophageal inflammation results from repeated exposure to food and aeroallergens in genetically susceptible individuals (38).

The documented cytokine expression profile in the esophageal tissue of EoE patients is that of a T_H_2 inflammatory response (13). The activated T_H_2 response leads to the recruitment and activation of eosinophils and mast cells, which degranulate, releasing products that instigate tissue damage and repair (39). Interestingly, T_H_1 cytokines including tumor necrosis factor (TNF)-α (expressed by esophageal epithelial cells) (39) and interferon (IFN)-γ (up-regulated by peripheral blood T cells) (40) are also found in increased numbers in esophageal biopsies (41). This may explain the non-IgE, type IV hypersensitivity (cell mediated) mechanism of EoE (14). It is postulated that the EoE-defining endoscopic and histologic manifestations are a culmination of the disease process (15) which, may have debilitating long-term effects including strictures and food impactions in untreated or poorly managed cases of EoE.

### Eosinophils

Eosinophils originate from CD34+ myeloid precursor cells in the bone marrow, mature to a granulated state and migrate to vascular spaces (39). They tend to be present in all layers of the esophagus in EoE, but predominate in the lamina propria and submucosal regions. Eosinophils contain many preformed granule proteins including eosinophil cationic protein (ECP), major basic protein (MBP) eosinophil peroxidase (EPO), and eosinophil-derived neurotoxin (EDN), which are released into tissues upon stimulation and degranulation (42). Additionally, eosinophils synthesize and release cytokines including IL-5, IL-13, transforming growth factor (TGF)-α and -β, chemokines (eotaxins and RANTES), and lipid mediators such as platelet activating factor (PAF) and leukotriene C4 (42). The process of eosinophil maturation and migration is stimulated by IL-5, IL-13, and granulocyte-macrophage colony stimulating factor (GM-CSF) (9). Eosinophil-derived angiogenic molecules may increase vascularity and facilitate inflammatory cell recruitment. TGF-β1 and matrix metalloproteinase 9 (MMP)-9 are fibrogenic mediators implicated in airway remodeling (42). Additionally, MBP and MMP-9 have been implicated in the disruption of esophageal epithelial integrity though their involvement in smooth muscles, fibroblasts, and cell-adhesion molecules (43). These processes may culminate in overall esophageal dysfunction through the consequent tissue remodeling.

Eosinophils are considered the main effector cells in fibrosis in a variety of hypereosinophilic syndromes and eosinophil-related allergic diseases including asthma and EoE (44). TGF-β (45) and eosinophilic granule proteins MBP and EPO (46) are the key eosinophil effector proteins. The importance of eosinophils in mediating tissue fibrosis is supported by evidence in both murine and human models (26). Interestingly, a recent study on fibrosis reversal with dietary and steroid therapy showed that improvement in esophageal eosinophilia and eosinophil degranulation within the epithelium was strongly associated with fibrosis reversal (47, 48) and symptom improvement. This finding is consistent with Kagalwalla et al. (45), who found improvements in epithelial remodeling in both dietary and corticosteroid therapy, and also found these improvements to be directly associated with improvement in esophageal eosinophilia (47). These findings not only highlight the importance of targeting fibrosis reversal in treatment of EoE, but also underline the importance of eosinophils in tissue remodeling.

### Eosinophil-derived angiogenic molecules

Angiogenesis, a characteristic of chronic inflammation, may facilitate inflammatory cell recruitment (49). Increased numbers of blood vessels are found in the lamina propria of pediatric patients with EoE than those with reflux esophagitis (50). The same study found an increased expression of the endothelial activation marker, vascular cell-adhesion molecule (VCAM)-1, compared with normal control subjects or patients with reflux esophagitis. Moreover, VCAM-1 expression positively correlated (*r* = 0.61) with esophageal eosinophil number, affirming its involvement in EoE. Increased VCAM-1 expression causes increased tethering of inflammatory cells to the endothelium of esophageal vessels, thereby facilitating eosinophil infiltration into the esophagus (51). Progressive tissue damage may result from this increased inflammatory cell recruitment facilitated by angiogenic products.

### Eosinophil-derived TGF-β1

Eosinophil-derived TGF-β1 has been implicated in the activation, proliferation, and synthesis of extracellular matrices of epithelial cells (39). Evidence suggests that TGF-β1 induces epithelial basal zone hyperplasia, contributing to esophageal wall thickening resulting in luminal narrowing (12). It also induces fibroblast activation and differentiation into myofibroblasts, with consequent over-production of extracellular matrix (ECM), predisposing to subepithelial fibrosis with consequent features of strictures and food impactions (52). Finally, TGF-β1 may induce smooth muscle hypertrophy, leading to thickening of the esophageal muscularis propria (53).

Accordingly, TGF-β1 would be a good therapeutic target in EoE. However, being a T-regulatory molecule, it is involved in the regulation of normal immune systems and is vital in repair (39). Consequently, the difficulty would lie in the localized delivery of a drug targeting TGF-β1 to the esophagus alone, without interfering with its normal functions in other tissues.

### MBP and MMP-9

Major basic protein levels correlate with basal cell hyperplasia (46) and has been associated with loss of barrier function in the esophageal epithelium (42). MBP has consequently been proposed as a player in subepithelial fibrosis in EoE (54). It appears to function by up-regulating the expression of fibroblast growth factor (FGF)-9, a molecule involved in epithelial homeostasis and proliferative response to injury (11). While initially being uncharacterized in the esophagus, FGF-9 has now been found in biopsy specimens of patients with EoE (55), hence the interest in its up-regulation by MBP. Another function of this molecule is smooth muscle contraction through its action on muscarinic M_2_ receptors, which may contribute to the dysphagia experienced by many patients with EoE (56).

A molecule with similar function to MBP, MMP-9 can be generated by structural and inflammatory cells and has the ability to secrete and activate latent matrix-bound growth factors (42). MMP-9 can thus degrade proteoglycans thereby enhancing airway fibrosis and smooth muscle proliferation (23). Although current research implicate MBP and MMP-9 in cell proliferation and tissue fibrosis, it must be highlighted that these molecules may have an organ-specific function, in which case their effect on bronchial remodeling may not be able to be generalized to esophageal remodeling.

### Mast cells

Mast cells, like eosinophils, are derived from CD34+ progenitors in the bone marrow, with their differentiation being regulated by surface c-kit receptor (CD117) (57). Mast cells contain several preformed mediators including histamine, cytokines, serine proteases (tryptase, chymase), and proteoglycans that are stored in cytoplasmic granules (58). They have a central role in innate immunity especially in allergic diseases, being the predominating cells in IgE-mediated responses. Attwood and colleagues (3) first discovered the presence of mast cells in the EoE inflammatory infiltrate. Due to the suggested immunoallergenic nature of EoE, the study of the involvement of mast cells in this disease has recently increased (59).

The role of mast cells in EoE has been supported by both human and animal studies. Several studies have identified increased mast-cell numbers in patients with EoE (46, 60, 61). Further, epithelial mast-cell infiltration was noted to precede eosinophil accumulation in a guinea pig model of EoE (62). Mast-cell numbers were also significantly increased after intranasal exposure to cockroach and dust mite allergen in a murine model of EoE (63). Importantly, studies on humans with EoE have found a correlation between mast-cell counts and characteristic features of EoE including intraepithelial eosinophil numbers, basal zone hyperplasia, and eotaxin-3 level (30, 54).

The immunoregulatory function of mast cells has been increasingly recognized in EoE (64). Through its action on H2 and H4 receptors, mast-cell-derived histamine has been shown to modulate immune responses by acting on dendritic cells and T-lymphocytes (65). It may act to maintain a favorable environment for allergic immune responses by recruiting T-lymphocytes, enhancing the proliferation of eosinophils in the bone marrow and inducing B-cell class switching to IgE production (66). Additionally, mast-cell-derived proteoglycans such as heparin are known to potentiate eotaxin-induced eosinophil recruitment *in vivo* (67), highlighting a key combinatory role amongst eosinophils and mast cells in EoE pathogenesis.

Mast cells may also have an effector function through specific enzymes. Mast-cell-specific genes encoding for proteins including chymase, tryptase, and carboxypeptidase A3 are significantly unregulated in EoE (30). These proteins are known to increase mucus secretion and smooth muscle contraction in the bronchi of asthmatics (39). Furthermore, Chehade and colleagues (54) found increased expression of tryptase-positive mast cells that produced TGF-β contributing to tissue remodeling. Additionally, mast cells have been found in the muscle layers of the esophagus and may cause contraction of muscularis mucosae through histamine-activated acetylcholine resulting in trachealization of the esophagus, as observed endoscopically in patients with EoE (68).

Despite evidence supporting mast-cell involvement in EoE, there is currently no evidence supporting therapies targeting mast cells in EoE. Lucendo et al. (59) found no statistical decrease in mast-cell numbers in EoE patients following 3 months of glucocorticoid therapy (fluticasone propionate 500 μg b.i.d). In the same study however, there was a significant reduction in IgE positive cells. A randomized controlled trial found a significant decrease in mast-cell numbers in pediatric patients with EoE after 3 months fluticasone treatment with fluticasone (880 μg b.i.d) (69). However, 1-month treatment of EoE in a pediatric cohort, with the mast-cell stabilizer sodium cromoglycate (100 mg q.i.d), did not significantly affect symptoms or eosinophil counts (2). Overall, this suggests that although the involvement of mast cells is clear, its role in the pathogenesis of EoE is not yet fully characterized.

### The role of T_H_2 type inflammation in EoE pathophysiology

Serum IgE measurements demonstrate that the majority (80%) of patients with EoE have identifiable hypersensitivity to both food and aeroallergens (70). Inferentially, the majority of EoE cases may be associated with an IgE-mediated type 1 hypersensitivity reaction. Such reactions involve antigen presentation to CD4+ T-helper 2 (T_H_2) cells, which stimulate B-cell class switching to IgE production (39). This differs from T_H_1 responses where the immune system is directed toward a chronic intracellular infection, involving the activation of cytotoxic T cells and macrophages by T_H_1 cytokines such as IFN-γ. Most adaptive immune responses are a mixture of both T_H_1 and T_H_2 however, as suggested by the cytokine expression profile, EoE is a predominantly T_H_2-mediated condition (13). The following section discusses the contributions of B cells and T_H_2 cytokines such as IL-5 and IL-13 in the pathogenesis of EoE.

### B cells

In allergic contexts, T_H_2 cells stimulate B-cell (CD20+) class switching to produce IgE antibodies. IgE binds to its respective high-affinity receptor (FCεRI) present on the surface of mast cells, and this complex can then bind to an antigen, leading to mast-cell activation and degranulation (57). Unfortunately, despite this knowledge of B cells in allergic reactions, their role in EoE is poorly understood. One study found B cells to be significantly increased in the epithelium and lamina propria of EoE patients compared to healthy controls, and this value correlated significantly (*r* = 0.744) with mast-cell number (71), which in turn has been shown to correlate with eosinophil numbers (54). In Vicario and colleagues (71) study, evidence showed local B lymphocyte class switching to IgE expression (71). Another study found no change in B-cell numbers in untreated or corticosteroid-treated EoE patients (72). The possible discrepancy between the studies may be due to differences in patient demographics, and further such studies are required to substantiate the role of B cells in EoE.

### Cytokines

Interleukin-4 is responsible for the differentiation of naïve T-helper (T_H_0) cells into activated T_H_2 cells. IL-4 stimulates B-cell proliferation and maturation within plasma cells, regulates class switching of antibodies, and increases IgE production. Blanchard and colleagues (13) found no significant differences in the level of IL-4 between EoE patients and non-EoE controls, however IL-4 mRNA levels were significantly decreased in EoE patients following glucocorticoid therapy or elemental diets. In the same study, elevated IL-4 and IL-5 mRNA levels were found in allergic EoE patients compared to their non-allergic counterparts. Interestingly, there was no statistical difference in eotaxin-3 or IL-13 mRNA levels between the two patient groups. This may indicate a dysregulation of IL-4 and IL-5 in allergic EoE patients which possibly reflect their systemic allergic history rather than activity of the disease (13). However, this difference in cytokine expression between allergic and non-allergic EoE patients may open avenues for further research such as investigating possible difference in severity and response to treatments between the two patient groups.

Interleukin-5 functions in the proliferation, differentiation, and survival of eosinophils, T_H_0 cells, and mast cells (39). Mishra et al. (9) demonstrated a murine model for aeroallergen-induced esophageal eosinophilia by challenging mice with *Aspergillus fumigatus*. In this model, IL-5 was shown to be essential in aeroallergen-induced eosinophil recruitment to the esophagus (73). More recently, Blanchard et al. (13) found a significant increase in IL-5 mRNA in EoE patients compared to inactive EoE and healthy controls. The same study confirmed the importance of eotaxin-3 in the IL-5-dependent induction of esophageal eosinophilia as a 15-fold decrease in eosinophil number was found in eotaxin-deficient mice when subjected to the same aeroallergen challenge (73). IL-5 appears to function by enhancing eosinophil responsiveness to endogenous chemokines expressed by the esophagus such as eotaxin-3 (discussed later) (73).

Despite compelling evidence suggesting the importance of IL-5 in EoE, drugs targeting IL-5 have shown little clinical efficacy. A randomized controlled trial evaluating the anti-IL-5 antibody mepolizumab showed little clinical improvement, despite the decreased tissue eosinophilia compared to placebo controls (12). This questions current understanding of the mechanism by which IL-5 contributes to EoE, or that perhaps esophageal damage may prevail through other mechanisms, aside from that of IL-5-recruited eosinophils. Concordantly, reslizumab, another anti-IL-5 antibody reduced eosinophil counts in esophagi of children and adolescents, but symptom improvements were observed in all treatment groups and were not associated with changes in eosinophil number in the esophagus (74).

Interleukin-13, similar in structure and function to IL-4, may have a role in propagating the inflammatory response toward a T_H_2 mechanism, and has also been implicated in tissue fibrosis (39). Interestingly, IL-13-induced lung eosinophilia consequently induced esophageal eosinophilia as well (75), suggesting a possible intimate connection between respiratory and esophageal epithelia, or simply the systemic allergic reaction in both cases. Studies on bronchial remodeling in asthma, have shown that IL-13 is essential for tissue fibrosis and airway mucous production (52). Similar findings were seen in EoE, when Blanchard and colleagues (60) found a 16-fold increase in IL-13 mRNA in biopsies of pediatric patients with esophageal fibrosis, compared to non-EoE controls. IL-13 is also known to stimulate eotaxin-3 production by epithelial cells, which in turn stimulates eosinophil recruitment (60).

Additionally, IL-13 may stimulate fibroblasts to overexpress periostin and down-regulate filaggrin. Periostin is an ECM molecule that promotes eotaxin-induced eosinophil recruitment and regulates eosinophil adhesion (76). Filaggrin is a structural barrier protein in skin keratinocytes; down-regulation of this protein has been implicated in the impairment of esophageal barrier function and development of atopic dermatitis (76). Filaggrin is a member of the epithelial differentiation cluster (EDC), and together with involucrin (another EDC member), prevents the proteolytic destruction of keratin during differentiation of epidermal cells (77, 78). This forms an important barrier function in cornified epithelial cells, although its function in esophageal epithelial cells is less clear. A recent study on the effect of IL-13 on genes involved in epithelial differentiation concluded that IL-13 plays a large role in the up-regulation of genes such as Ki67 and down-regulation of EDC genes such as filaggrin and involucrin, which cumulatively contribute to the eotaxin-mediated recruitment of eosinophils to the esophageal epithelium. Not only does this open an avenue for further research on possible therapeutic strategies targeting IL-13 in EoE, but also suggests the importance of IL-13 in the pathogenesis of EoE.

Recently, Zhu et al. (79) found that IL-15 is involved in the induction of eosinophil-selective cytokines and chemokines by CD4+ T cells. This study found a 6- to 10-fold increase in the levels of both IL-15 and its receptor IL-15Rα in esophageal tissues, and a twofold increase in serum IL-15 protein levels in patients with EoE. Additionally, the level of esophageal eosinophilia in patients both treated and untreated EoE correlated significantly with the IL-15 transcript. Importantly, the *IL-15R*α-deficient mice were protected from the development of experimental EoE (79). Interestingly however, these mice were not protected from airway inflammation, suggesting that IL-15 many have an organ-specific mechanistic induction of esophageal eosinophilia in mice.

### Eotaxins

The three eotaxins are chemokines with selective eosinophil-chemoattractant activity and act on CCR-3 receptors (39). Blanchard and colleagues (30) found an increase in eotaxin-3 levels in esophageal biopsies of patients with EoE. In the same study, these levels correlated with esophageal eosinophil numbers. Konikoff and colleagues (80) found a correlation (*r* = 0.32) between eotaxin-3 protein level in peripheral blood and esophageal eosinophilia. Interestingly, eotaxin-1 and eotaxin-2 levels did not correlate. Battacharya and colleagues (81) however showed contradicting findings with increased eotaxin-1, -2, and -3 levels in EoE, but also no correlation between eotaxin-3 levels and tissue eosinophilia.

A recent genome-wide expression analysis identified *eotaxin-3* as the single most up-regulated gene in EoE (30). Furthermore, a single nucleotide polymorphism in the untranslated region of the eotaxin-3 gene is associated with EoE (30). Interestingly, this expression profile is not associated with GERD, a disease triggered by acid reflux, also associated with esophageal eosinophilia (57). Blanchard et al. (30) found a strong correlation between eotaxin-3 level and disease severity, determined by the degree of basal cell hyperplasia and numbers of eosinophils and mast cells. In the same study, EoE could not be induced in CCR-3-deficient mice (30). Additionally, IL-5 and IL-13 have shown to increase eotaxin-3 release by esophageal epithelial cells (81) suggesting the combined contributions of these molecules in esophageal eosinophilia.

Eotaxin-3 has been increasingly recognized as a more accurate biomarker for the diagnosis of EoE. Some patients with GERD also present with eosinophil numbers >15/hpf, thus raising questions regarding the accuracy of using peak intraepithelial eosinophil counts as a diagnostic feature of EoE (6). The same study concluded that increased numbers of cells staining for MBP and eotaxin-3 was more predictive of EoE than eosinophil counts alone. This suggests possible flaws in current diagnostic criteria, and highlights the importance of eotaxin-3 in EoE.

Combining this evidence, the importance of eotaxin-3 in esophageal eosinophilia is widely accepted. It is proposed that IL-13, produced by T_H_2 inflammatory cells under allergic contexts stimulates the production of eotaxin-3 by epithelial cells. IL-5 and eotaxin-3 may act synergistically to induce esophageal eosinophil infiltration, allowing eosinophilic inflammation to ensue. However, the reason for which this inflammatory process is restricted to the esophagus remains elusive. Regardless, this localized function of eotaxin-3 in EoE may be important in minimizing side-effects, if it were to be considered a future therapeutic target.

### Esophageal epithelium

The esophagus is composed of stratified squamous epithelium, protected by a variety of organ-specific molecules such as mucous and antibodies. Additionally, the epithelium is a potent reservoir of cytokines and lipid mediators, which normally functions to cleanse the epithelial surface (42). Disease develops when these functions are dysregulated and disrupted (49). In an allergen-induced T_H_2 response, molecules such as IL-13 stimulate esophageal epithelial cells to produce eotaxin-3 (30) which in turn leads to esophageal eosinophil recruitment. The resulting activation and degranulation releases mediators that cause tissue remodeling.

Epithelial to mesenchymal transformation (EMT) is a process whereby epithelia lose many characteristics including polarity, and acquire properties of mesenchymal cells, including motility and loose cell-adhesion (45). It facilitates the development of tissue fibrosis in many organs in response to injury, including the lungs (45). TGF-β and MBP released by eosinophils or damaged epithelium, may induce EMT and contribute to subepithelial fibrosis (82). The degree of EMT in patients with EoE has been shown to correlate with the amount of TGF-β1, eosinophil number, and amount of subepithelial fibrosis (82). Eosinophils can also directly induce the expression of factors relevant to EMT and fibrosis in epithelial cells, such as TGF-α, MMP-9, and others, through the secretion of MBP and cytokines such as IL-13 (45).

Blanchard and colleagues (30) suggested that intestinal epithelia are capable of releasing eotaxins, which are essential in eosinophil migration to the GIT. Epithelia also promote eosinophil survival, by releasing epithelial-derived GMSF, which delays apoptosis (42). Additionally, Hahn and colleagues (83) found that during allergic inflammation, there is an increased expression of nerve growth factor in airway epithelial cells, which may promote the survival of tissue eosinophils. However esophageal epithelial cells may have an organ-specific interaction with eosinophils, as supported in studies showing the necessity of eotaxin-3 in the induction of esophageal eosinophilia (30, 73).

Esophageal epithelial cells also express IL-4α, IL-13Rα1, and IL-13Rα2, which are all the components of the IL-13 receptor, rendering esophageal epithelial cells vulnerable to influence by IL-13. As mentioned previously, IL-13 plays a large role in the eotaxin-mediated recruitment of eosinophils to the esophageal mucosa, through the stimulation of epithelial cells to up-regulate some genes and down-regulate others. Interestingly, the same study found that biopsies of normal and EoE diseased esophagi, both showed an over-expression of eotaxin-3 in response to IL-13, suggesting that IL-13/IL-13 receptor/STAT6 pathway is similar in both normal and EoE patients (67).

## Conclusion

In summary, EoE is an inflammatory disorder (Table 1) that may be initiated by a hypersensitivity reaction to aero- or food allergens, with a late-phase characterized by eosinophil recruitment and subsequent tissue damage. It is currently characterized by excessive intraepithelial eosinophils, however emerging research shows that increased levels of eosinophil-degranulation products (MBP) and eotaxin-3 may be more accurate biomarkers of EoE. EoE has incompletely defined pathophysiology, having characteristics of mainly T_H_2 type immune responses but also includes some T_H_1 cytokines which appear to strongly contribute to tissue fibrosis with esophageal epithelial cells providing a hospitable environment for this inflammatory process.

**Table 1:** **Key findings and current treatments**.

**KEY FINDINGS**
EoE is an inflammatory disorder that is most likely initiated by a hypersensitivity reaction to aero- or food allergens, with a late-phase characterized by eosinophil recruitment and subsequent tissue damage
EoE predominates in the Caucasian male population. Mutations in the *TSLP gene* may provide a mechanism for the male predilection of EoE
Eosinophil-derived molecules including TGF-β1, MBP, and MMP-9 play a central role in fibrosis
Some mast-cell-derived molecules such as heparin potentiate eotaxin-mediated eosinophil recruitment to the esophagus, while others such as histamine may contribute to the abnormal functioning of esophageal musculature through its effect on enteric nerves
IL-5 was shown to be essential in aeroallergen-induced eosinophil recruitment to the esophagus, and IL-15 many also contribute to the same process
IL-13 plays a large role in the up-regulation of genes such as Ki67 and down-regulation of EDCs such as filaggrin and involucrin, which cumulatively contribute to the eotaxin-mediated recruitment of eosinophils to the esophageal epithelium
MBP and eotaxin-3 levels in esophageal biopsies may be more accurate diagnostic biomarkers of EoE
Esophageal epithelial cells are a potent reservoir for cytokines and express receptors such as IL-4α, IL-13Rα1, and IL-13Rα2, making them vulnerable to molecules such as IL-13
**CURRENT EFFECTIVE TREATMENTS FOR EoE**
Topical corticosteroids
Fluticasone propionate
Budesonide
Elimination diets
Elemental diets
Esophageal dilation to treat strictures

Eosinophil-degranulation products appear to play a central role in tissue remodeling in EoE. Of note, TGF-β1, MBP, and MMP-9 affect the esophageal epithelium by inducing EMT, interfering with function of esophageal musculature and cellular integrity. This remodeling and dysregulation predisposes to fibrosis. Mast-cell-derived molecules such as histamine may affect enteric nerves and may act in concert with TGF-β to disrupt the normal functioning of esophageal musculature. Additionally, the esophageal epithelium may facilitate the inflammatory process under pathogenic contexts such as in EoE. Collectively, these contribute to the characteristic histological features of EoE such as subepithelial fibrosis and esophageal thickening, leading to endoscopic findings of luminal narrowing, stricture formation, and trachealization, resulting in symptomatic presentations such as food impactions, dysmotility, and dysphagia. The overall inflammatory response is regulated and propagated with the assistance of eosinophil-derived angiogenic products facilitating recruitment of inflammatory mediators, and also the regulatory functions of molecules such as IL-13 and also mast cells.

The understanding of EoE is still in its infancy. While allergy is implicated, other mechanisms of EoE are also acknowledged in the literature, which implicate mainly eosinophil degranulation and eosinophil-mast-cell cross talk, in disease pathogenesis. While future research may identify possible therapeutic targets, localizing the delivery of these therapies to the esophagus alone remains a challenge. Furthermore, as discussed, emerging evidence suggests a genetic contribution to EoE. This is significant, as recent studies have demonstrated the coexistence of EoE with another genetically inherited condition, CD (27, 28). Further research is required to ascertain the effect of CD on the presentations and treatment outcomes of EoE.

## Conflict of Interest Statement

The authors declare that the research was conducted in the absence of any commercial or financial relationships that could be construed as a potential conflict of interest.
